# Functional Characteristics and Stress Tolerance of Microbiota in Botswana’s Traditional Sourdoughs

**DOI:** 10.1007/s00284-026-04880-8

**Published:** 2026-04-17

**Authors:** Thandiwe Semumu, Nerve Zhou, Kebaneilwe Lebani, Thando Ndlovu, Kabo Wale, Daniel Loeto

**Affiliations:** 1 Department of Biological Sciences, Faculty of Science, University of Botswana, Private Bag, 0022 Gaborone, Botswana; 2 Department of Biological Sciences and Biotechnology, Faculty of Science, Botswana International University of Science and Technology, Private Bag 16, Palapye, Botswana

## Abstract

Sourdough fermentation is a traditional biotechnological process used to improve the nutritional value, flavour profile, and textural attributes of baked goods. Sourdough principal microflora is represented by yeasts, lactic acid bacteria (LAB) and acetic acid bacteria (AAB). The yeasts are primarily responsible for leavening whereas the LABs and AABs carry out the acidification of the dough and both contribute to the flavour of the resulting bread. Our previous work explored the microbial species diversity of sourdoughs in Botswana and showed the unique microbial communities involved in sourdough production from different areas in Botswana. This study is aimed at characterizing yeasts and bacterial isolates from 9 traditional sourdoughs indigenous to Botswana. Here we report the functional characterization of 21 yeasts, 9 LAB, 4 AAB, and 11 other bacteria isolated from traditional sourdough as reported in our previous study. These isolates were characterized based on their ability to assimilate different carbon sources found in flour (maltose, glucose, sucrose, fructose, raffinose and maltotriose), capability to ferment carbon sources (maltose, glucose, sucrose and fructose) in flour and ability to withstand baking associated stresses (thermal stress, osmotic stress, oxidative stress and ethanol stress). This characterization aimed to compare them with conventional baker’s yeast used in modern bread making and to assess their potential for future commercialization. Our results show that the majority of isolated *S. cerevisiae* strains can utilise different carbon sources, ferment them and withstand baking associated stresses. These are key baking traits poorly expressed in the conventional baker’s yeast. In contrast, the isolated LAB and AAB exhibited limited carbon source utilization. Despite the noted poor utilization of these isolates, we noticed that *L. plantarum* (ME1-B1), *B. cereus* (GT1-B1) and B. *zhangzhouensis* were able to withstand baking associated stresses better than all other bacteria isolates. This study shows how isolated *S. cerevisiae* strains and non-conventional yeasts have comparable capability to the conventional baker’s yeast, highlighting their potential as sourdough starters and prospects for future commercialization.

**Supplementary Information:**

The online version contains supplementary material available at 10.1007/s00284-026-04880-8.

## Introduction

Traditional sourdough is primarily a mixture of flour and water that undergoes fermentation by active lactic acid bacteria (LAB), acetic acid bacteria (AAB) and yeasts, which multiply and establish varying levels of dominance [1]. The process involves grinding cereals, or legumes and adding water to create dough, which then transforms into sourdough over time [2]. The ripe sourdough microflora is formed by stable associations of lactobacilli, acetic acid bacteria and yeasts that may persist for years. Traditional sourdough has been utilized for centuries in the production of fermented baked goods and as an alternative to baker’s yeast and chemical leavening agents. Numerous studies have demonstrated extensively that over 50 species of LAB, primarily from the *Lactobacillus* genus, and more than 20 species of yeast, predominantly from the *Saccharomycetaceae* complex, participate in the fermentation process [2, 3]. During the fermentation process, the yeasts are primarily responsible for leavening whereas the LAB and AAB carry out the acidification of the dough and both contribute to the flavour of the resulting bread [4]. Fermentation of LAB, AAB and yeast is a multi-day procedure to reach a pH value of below 4.5. The most common LAB species found in sourdoughs are *Lactobacillus acidophilus (L. acidophilus), L. farciminis, L. delbrueckii* (obligate homofermentative), *L. casei, L. plantarum, L. rhamnosus* (facultative heterofermentative), *L. brevis, L. sanfransicencis, and L. fermentum* (obligate heterofermentative) whereas universal sourdough yeasts appear to be *Saccharomyces cerevisiae, Candida milleri,* and *Kazachstania humilis* [5]*.* Common AAB species found in sourdoughs are *Acetobacter pasteurianus, Acetobacter indonesiensis* and *Acetobacter malorum* [6, 7].

Sourdough is characterized by its acidic pH, unique aroma profile and an increased volume as a result of gas formation [2]. High volume, soft and elastic crumb structure, good shelf life, stress tolerance and microbiological safety of the product are the most important attributes when characterizing the quality of leavened wheat breads [8]. The utilization of sourdough starter cultures as a natural leavening agent offers several advantages over conventional baker’s yeast such as; improved textural properties, enhanced flavour profiles, improved nutrition, superior sensory attributes of the final product [9, 10] as well as retardation of the staling process.

Yeast and bacterial strains are widely used in industries that rely on fermentation, such as baking, winemaking, brewing, and biofuel production, among others [11]. However, subjecting these yeasts and bacteria to harsh industrial conditions, which may include elevated temperatures, osmotic stress, oxidative stress, and inhibitory compounds, poses a significant challenge for industrial fermentation. These stresses have a notable impact on cellular macromolecules, resulting in growth inhibition, compromised survival, and ultimately, diminished fermentative activity [12]. Consequently, the reduced fermentative capacity of yeasts and bacteria leads to decreased productivity, rendering production processes inefficient and unsustainable. Therefore, utilisation of industrial strains with robust stress tolerance capabilities and attractive baking characteristics is viewed as a cost-effective approach that facilitates economically feasible bioprocessing.

This study was aimed at characterizing yeasts and bacteria isolated from traditional sourdoughs of Botswana with a focus on fermentative capabilities, utilization of carbon sources and ability to withstand baking associated stresses in order to assess their potential as functional sourdough starters. Here, we report the functional characterization of 19 yeasts, 9 LAB, 3 AAB and 11 other bacteria isolated from traditional sourdoughs. This work is a continuation of our previous study in which these strains were reported. The functional traits were compared to isolates from a commercial sample sourced from the United Kingdom as reported in our previous study. The comparator sample had 2 yeasts, 2 LAB and 1 AAB [13]. All microbial isolates were characterized based on their ability to assimilate different carbon sources, fermentative capabilities and ability to withstand baking associated stresses. This study confirms that sourdough yeasts and bacteria exhibit broad physiological diversity, actively assimilating diverse carbon substrates under different conditions of baking-related stresses.

## Materials and Methods

### Microbial Strains

A total of 19 yeast strains, 9 LAB, 3 AAB and 11 other bacteria isolated through culture-based methods on selective media and identified by molecular techniques (16S rRNA gene sequencing for bacteria and ITS region sequencing for yeasts) from sourdoughs around Botswana were used in the current study and were described in our previous study [13]. A commercial sample from the UK (brand withheld) was used as a comparator and it contained 2 yeasts, 2 LAB and 1 AAB [13]. The collection and identification of sourdough starters, as well as the techniques used for isolating and identifying yeasts and bacteria, were reported in the previous study [13]. A control conventional baker’s yeast (*S. cerevisiae)* from a local retail outlet sold as dry instant yeast (Anchor Yeast Co, South Africa) was used as a positive control.

### Culture Media

The yeast isolates were revived from − 80 °C freezers by plating them on yeast extract-peptone-dextrose (YPD) agar (Sigma-Alrich, USA) consisting of 2% glucose, 0.5% yeast extract and 1% peptone at a pH of 6.2. The yeasts were restreaked onto YPD agar and incubated (Thermo Scientific, MaxQ 6000, Ohio, USA) for 72 h at 30 ˚C. Thereafter, single colonies were selected for stress tolerance testing and functional characterization. For bacteria, isolates from − 80 °C freezer were plated on de Man, Rogosa and Sharp agar (MRS) (Sigma-Alrich, USA) at a pH of 6.4 ± 0.2 under microaerophilic conditions and incubated at 37 ˚C for 48 h for optimum growth. These bacterial isolates were also used for stress tolerance testing and functional characterization.

### Investigation of Carbon Assimilation

To assess carbon source utilization, yeast and bacterial isolates were cultured in yeast nitrogen base (YNB) (Sigma-Aldrich, USA) and minimal salt media (Sigma-Aldrich, USA), respectively and viewed under the microscope to ensure purity. Each medium was supplemented with a range of different carbon sources. The following sugars (all at 2%, w/v) were used as sole carbon sources in the assays: glucose, fructose, maltose, sucrose, raffinose and maltotriose. The yeasts isolates were first incubated overnight in 5 mL test tubes at 30 ºC in YNB broth with 5% glucose whereas bacterial isolates were incubated at 37 ºC in minimum salt media. The yeast cells were then harvested by centrifugation at 15 000 × g for 2 min whereas bacteria isolates were centrifuged at 10 000 × g for 10 min. The pellets were re-suspended in phosphate-buffered saline (PBS) (Sigma-Aldrich, USA) and starved for 8 – 12 h in PBS. For yeasts, the cells were diluted and inoculated into 100 μL of YNB media with the different sugars (5%, w/v) in sterile round bottom 96-well plates (Thermo Fischer Scientific, Dreieich, Germany) to a final optical density (OD) of 0.1 at 600 nm wavelength. A control conventional baker’s yeast (*S. cerevisiae)* from a local retail outlet sold as dry instant yeast (Anchor Yeast Co, South Africa) was used as a positive control. The plates were incubated for 12 h in a shaking incubator at 30 ºC (yeasts) and 37 ºC (bacteria) with initial and final OD readings taken using a Multiskan FC Microplate Photometer (Thermo Scientific, Dreieich, Germany). The final growth rate was compared to the initial growth rate by calculating the difference, to evaluate the change in cell biomass of the yeast isolates and bacteria in the presence of each sugar. Experiments were performed in biological triplicate, each carried out on a separate occasion. The heatmap of carbon assimilation of isolates was generated using the heatmap package in R, “rcomp” function, using raw data and default parameters [14].

### Analysis of Yeast Fermentative Capacity

An ideal baking yeast or bacterium possesses strong fermentative capabilities to efficiently leaven the dough. To investigate fermentative capability of the yeast strains, fermentation was carried out using yeast peptone (Sigma-Aldrich, USA) supplemented with 4 major carbon sugars commonly found in flour (maltose, glucose, sucrose and fructose) in 60 mL Luer-Lok™ syringes (BD® syringes) as described in our previous study [15]. A single colony from each yeast isolate was grown overnight in 2 mL of YPD in 5 mL culture tubes at 30 °C and placed on an incubator/shaker (Thermo scientific, Dreieich, Germany) set at 180 rpm. Then, 2 mL of the fermentation broth was collected through centrifugation at 15,000 × g for 2 min using a Heraeus Pico 17 microfuge (Thermo Scientific, Dreieich, Germany). The harvested pellet was subsequently washed twice with 2 mL of sterile PBS (pH 7), followed by another centrifugation step (15,000 × g for 2 min). The resuspension was used to inoculate 3 mL YP broth supplemented with 5% maltose, 5% glucose, 5% sucrose, or 5% fructose in syringes at an initial OD_600nm_ of 1 and placed on an incubator/shaker as above. Uninoculated media was used as a negative control. The movement of the plunger, triggered by the accumulation of CO_2_ in the syringes was recorded in millilitres (mL) every 2 h for a total duration of 24 h. The carbon dioxide production rate was calculated by determining the slope of the curve using the points at which the accumulation of CO_2_ was the fastest (the steepest slope). In addition, synthetic dough [16] was also used to investigate fermentative capabilities of the isolated yeasts under the same conditions as described for other carbon sources above. Statistical analysis was performed using GraphPad Prism (Version 9). Data is represented as the means of three biological replicates.

### Analysis of Bacteria Fermentative Capacity

To investigate fermentative capability of the bacterial strains, fermentation was carried out using de Man, Rogosa and Sharp broth (MRS) (Sigma-Aldrich, USA) supplemented with 4 carbon sources found in flour (maltose, glucose, sucrose and fructose) in 5 mL Luer-Lok™ syringes (BD® syringes). A single colony from each isolate was grown overnight in 2 mL of LB broth in 5 mL culture tubes at 37 °C and 180 rpm on an incubator/shaker (Thermo scientific, Dreieich, Germany). Fermentation broth was collected through centrifugation at 12,000 × g for 1 min using a Heraeus Pico 17 microfuge (Thermo Scientific, Dreieich, Germany). The harvested pellet was washed twice with 2 mL of sterile PBS (pH 7). The re-suspension was used to inoculate 1 mL MRS broth supplemented with 5% maltose, 5% glucose, 5% sucrose, or 5% fructose in syringes at an initial OD_600nm_ of 1 and incubated under 37 °C at 180 rpm on a shaker (Thermo scientific, Dreieich, Germany). The movement of the plunger, triggered by the accumulation of CO_2_ in the syringes was recorded in millilitres (mL) every 2 h for a total duration of 24 h. Experiments were performed in biological triplicate, each carried out on a separate occasion. Statistical analysis was performed using GraphPad Prism (Version 9).

### Assessment of Microbial Isolates to Stress Tolerance

Tolerance to various baking-associated stresses was investigated to determine the utility of different yeasts and bacteria in baking. To test for ability to withstand baking associated stresses, a single yeast and bacteria colony were grown overnight in 2 mL of YPD and MRS broth respectively in 5 mL culture tubes. The tubes were shaken at 180 rpm at 30 °C (yeast) and 37 °C (bacteria) on a shaker (Thermo scientific, Dreieich, Germany). Then, 2 mL of the fermentation broth was collected through centrifugation at 15,000 × g for 2 min for yeasts and 10,000 × g for 10 min for bacteria using a Heraeus Pico 17 microfuge (Thermo Scientific, Dreieich, Germany). The harvested pellet was subsequently washed twice with 2 mL of sterile PBS (Sigma-Aldrich, USA) (pH 7), followed by another centrifugation step (15,000 × g for 2 min) and resuspension in PBS. In 98-well plates, YNB broth and minimal salt media were only supplemented with glucose (5%) as the sole source of carbon for every stressor. The stressors investigated were ethanol stress (2%, 4%, 6%, 8% and 10%), oxidative stress using hydrogen peroxide (1 mM, 2 mM, 3 mM, 4 mM and 5 mM), osmotic stress using sodium chloride (NaCl) (0.5 M, 0.75 M, 1 M, 1.25 M, 1.50 M and 1.75 M), acidity using hydrochloric acid (1%) and alkalinity using 1% sodium hydroxide. Thermal stress tolerance was assayed over a range of incubation temperatures from 25 – 42 °C. The plates were incubated in a shaking incubator at 30 ºC (except when temperature was the stressor being investigated) for 12 h with initial and final OD readings taken using a Multiskan FC Microplate Photometer (Thermo Scientific, Dreieich, Germany). The stress tolerance ranges were selected based on previously reported tolerance levels of yeasts and lactic acid bacteria (LAB) in similar studies [8]. The initial growth rate was subtracted from the final growth rate to compare the change in cell biomass of the yeast and bacterial isolates in the presence of each stressor. Experiments were performed in biological triplicates, each carried out on a separate occasion. The heatmap of stress responses of microbial isolates was generated using the heatmap package in R-studio [17], using raw data and default parameters, whereas principal component analysis (PCA), was generated using the R package, “rcomp” function [14]. For both the heatmap and PCA analyses, raw data was first inspected for low-variance features, which were removed to reduce noise. The data was then normalised to ensure comparability across variables.

### Statistical Analysis

To test if the isolates from traditional sourdough and the control baker’s yeast has significantly different fermentative capability, analysis of variance (ANOVA) at a 0.05 significance level to determine overall differences. When significant differences were observed (p < 0.05), mean values were compared using Tukey’s test at the 0.05 significance level to assess pairwise comparisons between isolates based on their fermentative capabilities. To provide an integrated visualization of overall stress resilience, the compiled results were presented using a heatmap and principal component analysis (PCA) plots.

### Carbon Utilization Profiles of Sourdough Yeast and Bacterial Isolates

**Fig. 1:**
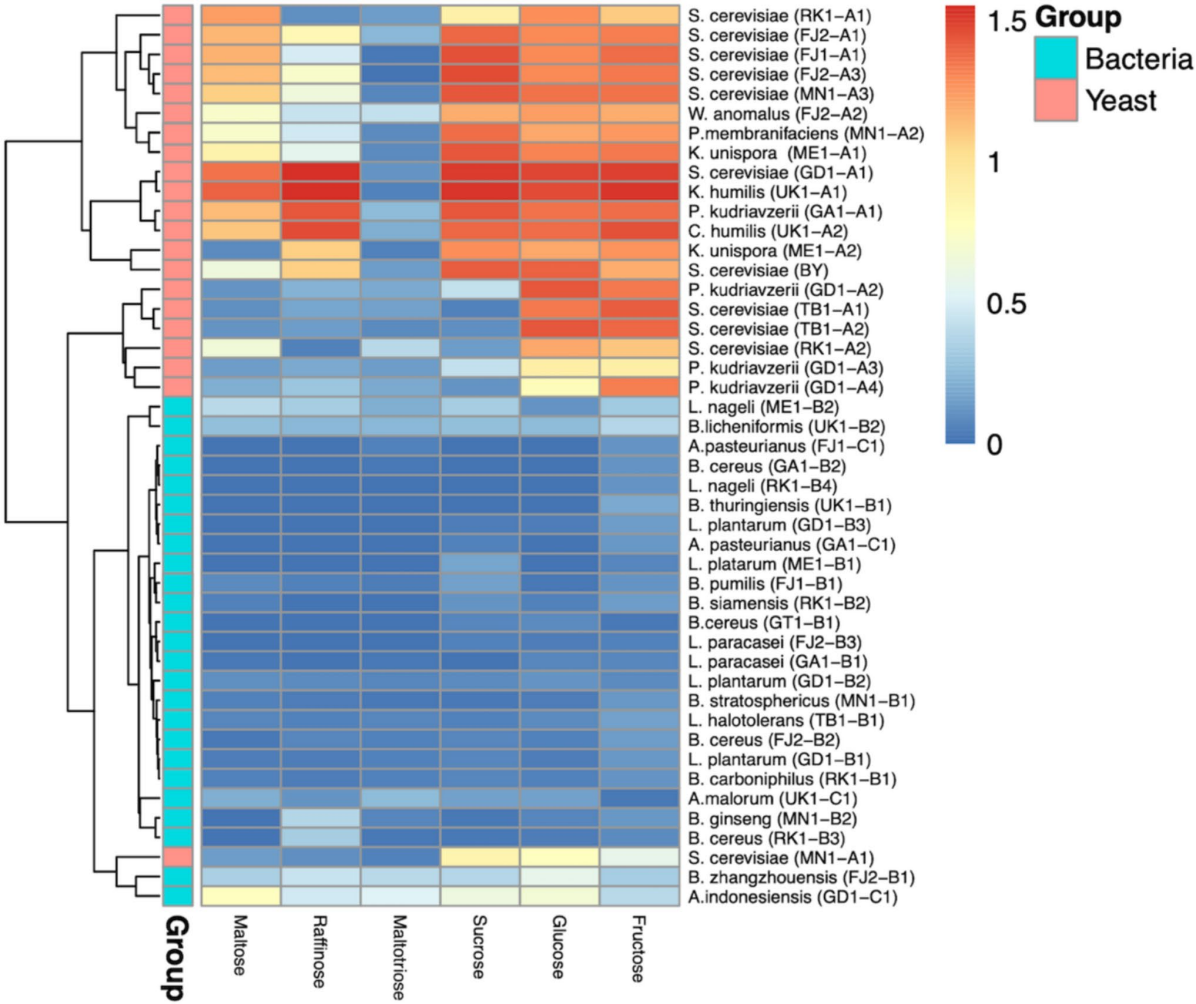
A heatmap depicting carbon assimilation ability of isolated yeasts and bacteria from traditional sourdough collected in various areas of Botswana. The rows correspond to the 19 isolated yeasts together with the control baker’s yeast, 9 LAB, 3 AAB and 11 other bacteria together with 2 yeasts, 2 LAB and 1 AAB from the UK comparator sample while the columns correspond to the six different carbon sources used. The growth scores range from 0 to 1.5; 0 = no growth, 0.5 = poor growth, 1 = average growth, 1.5 = good growth. The maximum possible score of 1.5 is given in orange while the minimum possible score of 0 is given in blue. The yeasts and bacteria were categorized based on well they perform in different carbon sources and close relations amongst each other. Data is represented as the means of three independent biological replicates

The ability to assimilate and ferment diverse carbon sources is an important trait in sourdough cultures. A total of 19 yeast isolates, 9 LAB isolates, 3 AAB, 11 other bacterial isolates and comparator strains from traditional sourdough of Botswana (See Supplementary material **S1-S4**) as described by Semumu, Zhou [13] were assessed for their ability to utilize different carbon sources found in flour (maltose, glucose, fructose, sucrose, maltotriose and raffinose) (Fig. 1**).** The isolates exhibited preferential fermentation of certain carbon sources over others. Most yeasts showed robust fermentation of sugars such as glucose, sucrose and fructose, producing gas rapidly, whereas more complex sugars like maltose, maltotriose and raffinose were utilized more slowly or not at all. Among the isolated yeasts, *Saccharomyces cerevisiae* (GD1-A1) and *Kazachstania humilis* (UK1-A1) exhibited the highest ability to utilize glucose, fructose, maltose, raffinose and sucrose as compared to all other yeasts isolated and the control baker’s yeast (*Saccharomyces cerevisiae* (BY)) whereas *Bacillus zhangzhouensis* (FJ2-B1) and *Acetobactor indonesiensis* (GD1-C1) exhibited the highest ability to utilize different carbon sources as compared to all other isolated lactic acid bacteria and acetic acid bacteria. See supplementary material **S5** for more details.

### Fermentative Capabilities of Yeasts from Traditional Sourdoughs of Botswana

**Fig. 2:**
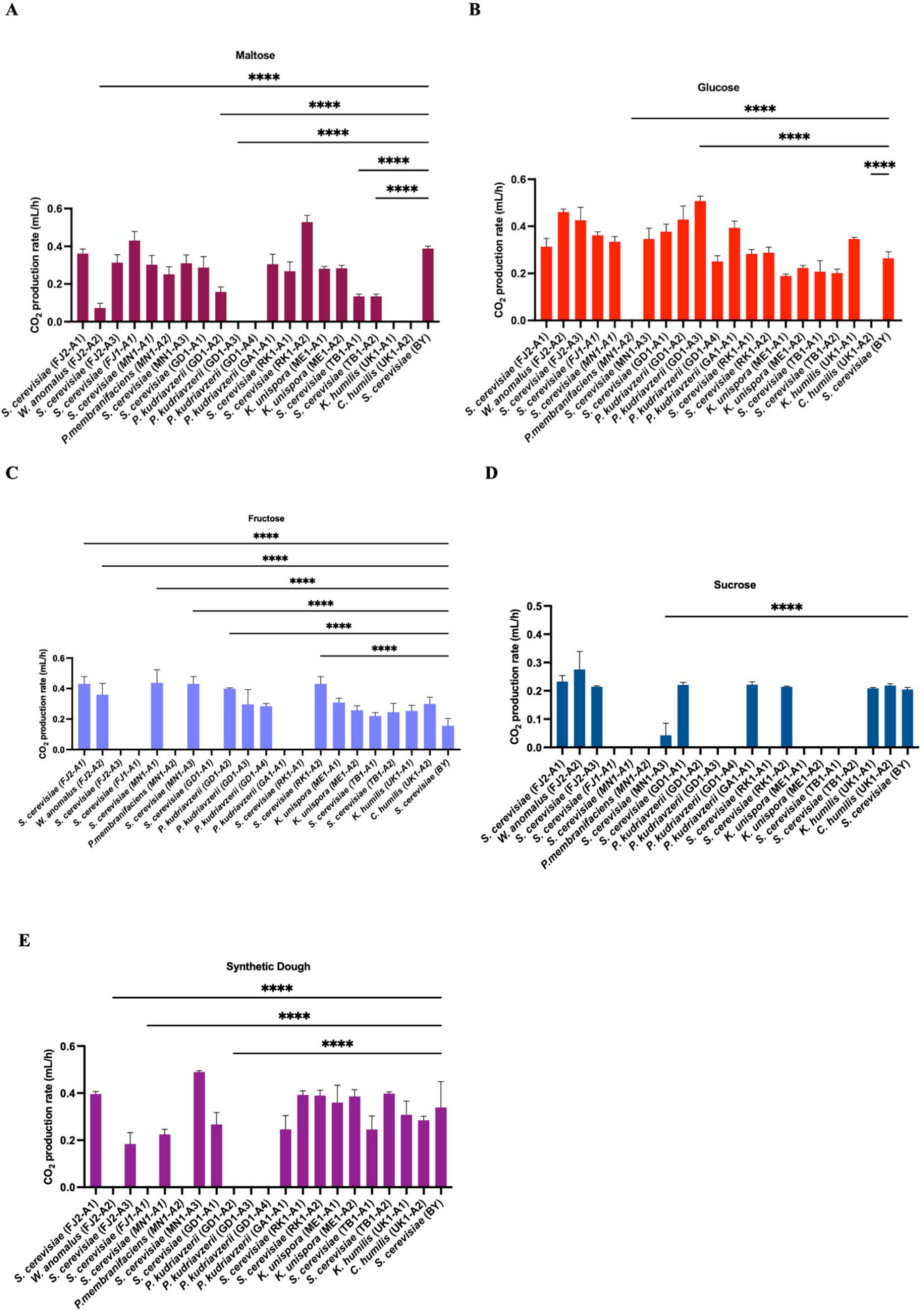
Carbon dioxide production rate of isolated yeasts using different carbon sources in comparison to the control baker’s yeast. **A**. maltose, **B**. glucose, **C**. fructose, D. sucrose, **E**. Synthetic dough. 21 isolated yeasts including comparator UK isolates fermentation with different carbon sources. The isolates showed elevated fermentation capabilities in different carbon sources within 24 h (profile of fermentation after 24 h was excluded for brevity). This experiment was done in triplicate and repeated thrice **** indicates *P* < 0.0001, as determined by one-way ANOVA and Tukey test. Only data with a *P* < 0.0001 was represented on the figure. Error bars represent standard error of the mean. See supplementary material Table S7 for full statistical analyses

In addition to investigating the microbial isolates’ capacity to assimilate diverse carbon sources, the yeast isolates were further assayed for fermentative capabilities based on carbon dioxide production rate. While carbon assimilation is important for biomass production and production of some volatile organic compounds, the ability to ferment the carbon sources at a faster rate is more crucial as it contributes to dough leavening and production of more aroma compounds [8, 15]. Our results show fermentative potential of yeast isolates from sourdough using different carbon sources found in flour (Fig. 2). Our results show that 85% (18/21) yeasts isolates could ferment maltose and 90% (19/21) could ferment glucose compared to other carbon sources (sucrose and fructose). Our results also show how the isolates were comparable in activity levels to the control baker’s yeast (*S. cerevisiae* (BY)) that is monopolized in today’s market. See supplementary material **S6** for more details.

### Fermentation Capability of Bacteria from Traditional Sourdough of Botswana

The LAB strains are widely utilized in the food industry for fermentation. In this study we examined fermentative capabilities of isolated LAB and AAB in different carbon sources found in flour (maltose, glucose, sucrose and fructose). Here we note the fermentative potential of bacteria isolates from sourdough **(Supplementary material S3**). Generally, most isolated sourdough LAB and AAB could not significantly raise the plunger to show carbon dioxide production. However, some isolates only produced bubbles.

### Sourdough Yeast and Bacteria Exhibit Multiple Stress Tolerance

**Fig. 3:**
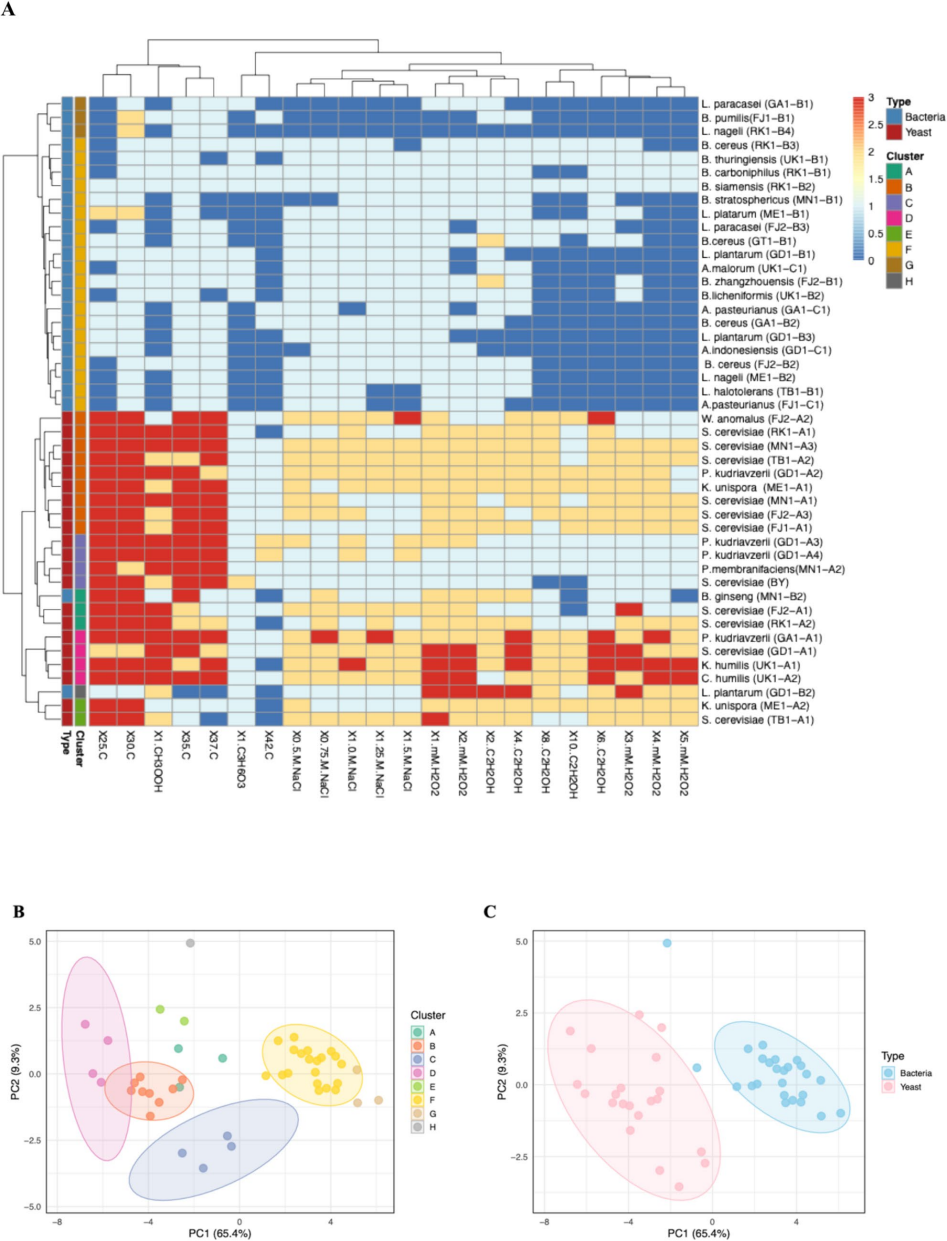
Stress tolerance of isolated yeasts and bacteria from Traditional sourdough of Botswana. **A**. heatmap showing stress tolerance (oxidative stress tolerance, ethanol stress tolerance, osmotic stress tolerance and thermotolerance) for both yeasts and bacteria **B**. PCA plot showing species distribution based on similarities and differences of functional characteristics **C**. PCA plot showing species distribution of yeasts and bacteria. The rows in the heatmap correspond to the 19 isolated yeasts together with the comparator isolates (UK isolates), 9 LAB, 3 AAB and 11 other bacteria while the columns correspond to baking associated stressors. The growth scores range from 1 to 3; 1 = no growth, 2 = average growth, 3 = good growth. The maximum possible score of 3 is given in orange while the minimum possible score of 1 is given in blue. Data are represented as the means of three independent biological replicates

Next, we sought to test whether our yeast and bacterial isolates could withstand stress encountered during fermentation, leavening and long-term storage, such as osmotic pressure, oxidative stress, different temperatures, and varying ethanol levels. Our study demonstrates that the isolates exhibited diverse stress tolerance capabilities. To visualize overall stress resilience, the compiled results were presented as a heatmap (Fig. 3A**)** and PCA plots (Fig. 3B and 3C). In the heatmap, the isolates were clustered using hierarchical clustering based on the pairwise Euclidean distances of their tolerance to specific stressor types. This approach groups isolates with similar response patterns by iteratively joining the closest pairs until a hierarchical tree is formed. The tree was then cut into eight distinct clusters (A–H), allowing isolates to be grouped according to similarity in their profiles. For the PCA plots, principal component analysis (PCA) was employed to reduce the dimensionality of the dataset. The first two principal components were used to visualize sample separation, where clustering patterns either reflected the eight groups defined by hierarchical clustering (Fig. 3B**)** and the broader classification into bacteria and yeast (Fig. 3C**)**. When coloured by the eight clusters, the PCA clearly reflects the groupings seen in the heatmap, while colouring by type (bacteria vs. yeast) shows an additional layer of separation between the two major isolate categories. Taken altogether, the heatmap clustering and PCA provide complementary views of how isolates are grouped based on their underlying data structure. See Supplementary material **S7** for more detailed information.

## Discussion

The capacity to utilize and ferment a variety of carbon sources is a key trait in sourdough making. From this study, all yeast isolates exhibited high specific growth rates on glucose and fructose, while the specific growth rate on maltotriose was notably lower (Fig. 1). Although maltotriose could be abundant in flour, it is much more complex than glucose and fructose and would require extracellular enzymes such as isomaltase to be available for the yeasts [18]. Among the yeast isolates, *Saccharomyces cerevisiae* strains from various locations dominated in their capability to utilize a wider range of carbon sources at high growth rates. *Pichia kudriavzevii* (GA1-A1) demonstrated the highest similarity to conventional yeast species, followed by *K. humilis* (UK1-A1) and *C. humilis* (UK1-A2), indicating the promising potential of non-conventional yeast species in the baking industry as potential sourdough starters. *S. cerevisiae* has been long domesticated and well adapted to the baking environment [19]. It is important to note that these isolated yeasts strains were well comparable to the baker’s yeast control strain. In comparison to previous studies, the performance of *S. cerevisiae* aligns with established findings, particularly regarding its preferential utilization of glucose and fructose. For instance, a study by Pinu, Edwards [20] demonstrated that *S. cerevisiae* EC1118 preferentially consumed glucose and fructose over other sugars during fermentation.

Yeast isolates obtained from the Francistown (FJ1-A1, FJ1-A2 and FJ2-A3) and Ratholo (RK1-A1) samples demonstrated a wide carbon assimilation range as they showed rapid growth in glucose, fructose, sucrose and maltose. *Pichia kudriavzevii* from GD1-A3 and GD1-A4 exhibited poor maltose assimilation, and this could be suggestive of possible reliance on a synergistic relationship with other associated microorganisms. Synergistic associations of microbial consortia in sourdough can alter metabolic pathways during fermentation [21]. A study by Caballero, Olguín [22] highlighted that although *S. cerevisiae* strains have enzymes for maltose uptake, the consumption rate might be slow. The isolates which showed the widest carbon assimilation and highest growth rates were from Tsabong (TB1-A1, TB1-A2), Gaborone (GA1-A1 and GD1-A1) as they exhibited high growth in glucose, fructose, sucrose, maltose and raffinose. Isolates from Maun (ME1-A1 and ME1-A2) had the least carbon assimilation range as they only showed high growth in glucose and fructose. Considering that microbial isolates from the same locations exhibited similar carbon assimilation ranges, this may indicate a biogeographical influence [13]. Additionally, other indirect geographical related factors such as preparation practices not only shape the biodiversity of sourdough microbiota but also influence their physiological capabilities [23, 24].

Sourdough starters represent a complex ecosystem, marked by intricate interactions among diverse yeast and bacterial species. Thoroughly isolating and characterizing these interactions presents a considerable challenge. The presence of LAB, AAB and other bacteria in sourdough cultures significantly influences the biodiversity and metabolic performance of the yeast isolates and the final bread. While co-culturing of yeast and LAB in sourdough has been shown to have no impact on yeast cell yield, co-fermentation significantly influences environmental factors such as availability of nutrients, synthesis of organic acids, pH reduction and changes of the rheological properties of sourdough [25]. Our findings revealed that most sourdough yeast and bacterial isolates can utilize a wide range of carbon sources, an important factor in sourdough production as readily available simple sugars are normally a limiting factor.

Gassing power (CO_2_ production rate) is another important attribute of a model baker’s yeast, which reduces the time taken to leaven dough, which is an important techno-economic factor [8, 15, 26]. Carbon dioxide is the primary byproduct of interest, focusing solely on its production in this study presents limitations, as it overlooks key metabolites such as organic acids and ethanol. In contrast, gas chromatography enables the detection of a broad spectrum of volatile compounds, providing a more comprehensive metabolic profile. We observed variations in the CO_2_ production rate, a key factor that influences the speed of dough leavening, with each isolate exhibiting a unique preference for its ideal carbon source. Generally, most sourdough yeasts preferentially ferment glucose and maltose as compared to fructose, synthetic dough and sucrose. *S. cerevisiae* (RK1-A2), *K. unispora* (MEI-A1) and *S. cerevisiae* (FJ1-A1) exhibited the highest fermentation capacity in the presence of maltose, while *P. kudriavzevii* (GD1-A4) and *P. kudriavzevii* (GD1-A3) did not show any significant fermentation of maltose because no gas production or visible growth was observed in maltose-containing media under test conditions, indicating an inability to ferment maltose. Even though *P. kudriavzevii* (GD1-A3) could not ferment maltose, the strain demonstrated high capability to ferment glucose along with *P. kudriavzevii* (GD1-A2), *W. anomalus* (FJ2-A2) and *S. cerevisiae* (FJ2-A3). All yeast isolates could ferment glucose and fructose, except for *P. kudriavzevii* (GA1-A1), *S. cerevisiae* (RK1-A1), *S. cerevisiae* (FJ1-A1) and *S. cerevisiae* (GD1-A1) which were not able to ferment fructose. The strain *S. cerevisiae* (MN1-A3) from Mokatse (a small village near Gaborone in Botswana) was among the isolates that could ferment all the sugars, and it demonstrated the highest fermentative performance in sucrose and synthetic dough. Isolates from Ratholo, Maun and Tsabong displayed higher fermentation capacity of synthetic dough highlighting their potential as starter cultures in baking and leavening products. The CO_2_ results were not entirely surprising, given the findings from the carbon assimilation tests. (Fig. 1) that showed that most isolated yeasts could utilize different carbon sources found in flour and so there was a higher chance of fermentation. Maltose is the most abundant carbon source found in flour [27], however *Pichia kudriavzevii* from Gaborone (GD1-A3 and GD1-A4) and United Kingdom (UK1-A1 and UK1-A2) preferred other carbon sources apart from maltose. This suggests that maltose, despite being the most abundant carbon source in flour, may not be the preferred carbon source for some yeasts [28].

On the other hand, LAB obtain energy through the process of sugar phosphorylation and are classified into two primary groups: homofermentative and facultative heterofermentative. Some bacteria can utilize both fermentation pathways and are referred to as facultative heterogeneous bacteria. Heterofermentative microorganisms are distinguished by their capacity to break down both hexoses and pentoses. They utilize the pentose phosphate pathway, which involves key enzymes such as ribose-5P epimerase and phosphoketolase. This pathway results in the production of lactate, carbon dioxide, and either ethanol or acetate as fermentation by products [29]. Carbon dioxide produced during this process is important for dough leavening, and the data generated by our study show varying patterns of carbon dioxide production by different microbial isolates. These patterns may be influenced by carbon source preferences and assimilation capabilities. Majority (14/21) of isolated bacteria could only produce bubbles when fermenting maltose, thus, not significant enough to raise the plunger. Similarly, for glucose fermentation, bubbles were observed for most (13/21) isolated bacteria, also not enough to lift the plunger. Also, for sucrose, fructose and synthetic dough fermentation, CO_2_ production by LAB and AAB was low. *L. plantarum* is a facultative heterofermentative strain well known for fermenting sugars to produce carbon dioxide, lactic acid, ethanol and acetic acid [30]. We noted that *L. plantarum* (GD1-B1, GD1-B2, GD1-B3and ME1-B1) could only produce carbon dioxide by producing bubbles when fermenting maltose and glucose. On the other hand, *L.nageli* showed ability to produce carbon dioxide in fructose fermentation. Fructophilic lactic acid bacteria (FLAB) are a newly identified group that includes certain species of *Fructobacillus* and *Lactobacillus*. Fructose is the optimal substrate for FLAB growth, and unlike other LAB, they exhibit very poor growth on glucose. Overall, the data suggests that our yeast isolates contributed more to dough leavening during fermentation as compared to LAB resulting from high CO_2_ production. While carbon dioxide is the primary byproduct of interest, other fermentation byproducts such as organic acids and volatile compounds, also play a significant role in shaping the characteristics of the final sourdough product. However, this study is limited to the analysis of carbon dioxide production.

Yeasts are known to perform most of the fermentation in sourdough as they are the main contributors to dough leavening [8, 15]. However, yeasts can experience many baking associated stresses at the onset, during and at the end of the fermentation processes. The tolerance of sourdough isolates to baking-associated stresses was assessed quantitatively. The sourdough environment is characterised by osmotic stress from the high sugar and salt concentration during the early stage of fermentation [31, 32]. The presence of salts can enhance microbial growth, however elevated concentrations (0.5 M, 0.75 M, 1 M, 1.25 M, 1.50 M and 1.75 M), may result in osmotic stress and ion toxicity. The sourdough associated yeast isolates exhibited tolerance to osmotic stress as they all showed growth at 1.5 M sodium chloride. Strains of *S. cerevisiae* (FJ1-A1), *P. membranifaciens* (MN1-A2), *K. unispora* (ME1-A2) showed less growth at 0.75 M to 1.5 M NaCl concentrations, indicating lower tolerance to osmotic stress compared to the other strains. The baker’s conventional yeast showed poor growth at all concentrations of sodium chloride used, as compared to all other isolated yeasts. This is in agreement with the findings of [8], where we reported the poor performance of conventional baker’s yeast at high sodium chloride concentrations. We have noted that our NaCl tolerance results align with previous sourdough studies. Notably *K. unispora* and *C. humulis* have been shown to have tolerance to high osmotic stress.

Oxidative stress tolerance was assessed, as yeasts encounter reactive oxygen species (ROS) during both dough fermentation and propagation [33]. In this study the sourdough associated yeasts isolates were exposed to varying concentrations of hydrogen peroxide to monitor their tolerance to oxidative stress. The yeast isolates exhibited diverse degrees of tolerance to oxidative stress with strains from Gaborone (GA1-A1 and GD1-A1) and UK (UK1-A1 and UK1-A2) having tolerance to high hydrogen peroxide concentration (up to 5 mM). The non-conventional *K. humilis* strain exhibited better tolerance to oxidative stress (5 Mm) as compared to other isolated yeasts. However, other non-conventional yeasts such as *P. kudriavzevii, P. membranificiens* and *W. anomalus* were among the least oxidative stress tolerant strains. These findings highlight the species-specific variability in stress tolerance among sourdough yeasts, emphasizing the need to consider individual strain robustness when selecting candidates for sourdough fermentation.

To overcome stress and thrive in different stressful conditions, LAB and yeasts have evolved a series of adaptation mechanisms [34, 35]. Metabolic versatility, such as preferential utilization of abundant sugars and production of fermentation metabolites, along with synergistic interactions between yeasts and bacteria, further support their persistence and functionality in the sourdough ecosystem. Apart from metabolism related stresses such as oxidative stress and osmotic stress, sourdough microorganisms are also exposed to environmental stresses such as thermal stress. Downstream processing for the preparation of biomass involving drying, storage and rehydration exerts thermal stress. Most of the yeast isolates could grow well at 37 °C except *S. cerevisiae* (TB1-A1) from Tsabong whereas only two *P. kudriavzerii* (GD1-A3 and GD1-A4) could withstand high temperatures of up to 42 °C which is known to be too high for yeasts to withstand. We observed that among the bacterial isolates, most isolates grew at 37 °C except for only 4 isolates (*B. thuringiensis* (UK1-B1), *B. stratosphericus* (MN1-B1), *L. plantarum* (ME1-B1 and *B. lichenformis* (UK1-)). The few strains that tolerated temperature up to 42 °C, were dominated by *Bacillus* spp. along with *L. paracasei* (GA1-B2) and *A. pasteurianus* (GA1-C1). Since sourdough is incubated at room temperature, Botswana’s elevated daily temperatures could result in elevated incubation temperatures for the dough. Furthermore, the fermentation process generates heat, thus sourdough microorganisms should tolerate elevated temperatures.

Our results show that the majority of the isolated sourdough bacteria can withstand different stressful conditions but at low growth rates as compared to yeasts. Both LAB and AAB isolates could not tolerate more than 3 mM of hydrogen peroxide. The strains that grew beyond that concentration were considered to have high tolerance to oxidative stress and they included *L. plantarum* (GD1-B2), *B. gingseng* (MN1-B2), *B. carboniphilus* (RK1-B1), *B. siamensis* (RK1-B2), *B. thurigiensis* (UK1-B1) and *B. licheniformis* (UK1-B2). Bacterial isolates from Mokatse, Gaborone, Ratholo and United Kingdom showed high oxidative stress tolerance. This may represent an adaptation to the sourdough environment, enabling them to survive and enhance the quality of the bread. Studies on *S. cerevisiae* and *L. helveticus* have shown that these microorganisms synthesize unsaturated fatty acids in their membranes which detoxify hydrogen peroxide and protect cells from oxidative stress [36].

As fermentation progresses, it results in accumulation of ethanol, thus the microorganism are expected to tolerate varying degrees of ethanol stress. The results (Fig. 3) show that majority of the yeast isolates were moderately tolerant to up to 10% (v/v) ethanol concentration. Of all the isolates tested, only S. cerevisiae (FJ2-A1) exhibited poor growth up to 10% (v/v) ethanol, with no growth observed at higher concentrations. Ethanol tolerance in bread making is an important attribute as it can prevent stuck fermentations and also helps reduce microbial contamination by antimicrobial activity. Our results further show that majority of the LAB and AAB isolates could not tolerate ethanol concentrations beyond 4% (v/v), with the exception of *L. paracasei* (FJ2-B3), *B. stratosphericu*s (MN1-B1), *L. plantarum* (GD1-B2), *B. ginseng* (MN1-B2), *B. carboniphilus* (RK1-B1), *B. siamensis* (RK1-B2), *B. cereus* (RK1-B3), *L. parabachnri* (ME1-B1), *L. plantarum* (GT1-B1), and *B. thurigiensis* (UK1-B1). The bacterial isolates with high ethanol stress tolerance were largely dominated by species of *Bacillus* genus. The same isolated LAB and AAB from this study showed a similar pattern when exposed to acetic acid and lactic acid tolerance of 1% (v/v) acetic acid and 1% (v/v) lactic acid. Bacterial isolates exhibiting higher tolerance to oxidative and ethanol stress, including *B. cereus* (FJ2-B1), *B. ginseng* (MN1-B2), *L. plantarum* (GD1-B1), *L. plantarum* (GD1-B2), *B. carboniphilus* (RK1-B1), *B. siamensis* (RK1-B2), *B. cereus* (RK1-B3), *B. thurigiensis* (UK1-B1), *B. licheniformis* (UK1-B2), *A. malorum* (UK1-C1) (Fig. 3A), also demonstrated tolerance to both 1% (v/v) acetic acid and 1% (v/v) lactic acid.

By utilizing principal component analysis (PCA), we were able to identify distinct stress tolerance profiles within the sourdough microbiota. We highlight that certain isolates exhibited high tolerance to heat but were sensitive to osmotic stress, while others showed broad resistance across multiple stress conditions, including ethanol and oxidative stress. These differences suggest potential functional diversity among strains, which may influence their suitability for specific fermentation applications. We notice **Group A** clustering isolates that are sensitive to almost all stressors under investigation and **Group B** forming a cluster with isolates that are sensitive to ethanol and oxidative stress. Strains exhibiting high osmotic or ethanol tolerance may be better suited to later stages of sourdough fermentation, where sugar and metabolite concentrations are elevated. Conversely, strains with higher thermal tolerance may be more resilient to process-related stresses such as baking conditions. Figure 3B provides a visual representation of these clusters, highlighting the diverse adaptations of these microorganisms. As expected, the yeasts formed a distinct cluster from bacteria based on their different roles and peculiar characteristics in sourdough processing (Fig. 3C). However, there were some outlier groups, suggesting diverse functions of traditional Botswana sourdough populations. In our previous study [13], we reported on the presence of potentially novel populations. Further research is needed to characterize these microorganisms and elucidate their contribution to the fermentation process of Botswana’s sourdough bread. Various complementary approaches such as gene expression and proteomics could be used in the future to further support the findings of the study. It is also notable that future studies should incorporate pH monitoring and control to better understand the effects of stressors on microbial performance during the production of sourdough.

## Conclusion

The present study revealed that the metabolic diversity of sourdough yeasts and bacteria enables them to assimilate a wide range of carbon substrates and generate a complex array of fermentation products. Despite *S. cerevisiae* strains showing higher fermentation capacity, non-conventional yeasts had comparable capability, demonstrating their potential as sourdough starters. Microbial diversity could be suggestive of a synergistic relationship between the bacteria and yeasts as an adaptation strategy to survive nutrient stresses unique to the sourdough environment. In addition to nutrient stresses, the sourdough environment is also associated with metabolism related stresses such as oxidative and osmotic stress as well as acidity stress from accumulation of lactic acid and acetic acid. Yeasts and bacteria isolated from sourdough showed high tolerance to baking associated stresses. Strains identified with desirable traits (e.g., high fermentation rate, high carbon assimilation capability and stress tolerance) can be standardized into commercial starter cultures. Such starter cultures can ensure consistent product quality, reduce fermentation time, and improve safety by suppressing undesirable microbes. The use of defined starter cultures can help industries scale up without compromising traditional quality. Stress-tolerant yeast and LAB strains identified in this study have potential for use as starter cultures in artisanal and industrial sourdough production, particularly under conditions involving thermal, osmotic, or ethanol stress. Additionally, their resilience suggests possible applications in developing robust fermentation processes suited to variable or resource-limited environments. The results indicate a potential relationship between the sample source and the physiological characteristics of sourdough microbiota. Isolates derived from the same sample demonstrated comparable metabolic performance, suggesting they occupy a shared ecological niche. However, more intensive studies with extensive sampling would be required to ascertain this observation. Furthermore, we recommend metagenomic and metatransciptomic studies to fully understand the interaction between these diverse microorganisms in the sourdough environment.

## Supplementary Information

Below is the link to the electronic supplementary material.

Supplementary file1

## Data Availability

Data is provided in the supplementary material.
