# Supplementary material for: Functional Characteristics and Stress Tolerance of Microbiota in Botswana’s Traditional Sourdoughs
Source: Curr Microbiol. 2026 Apr 17;83(6):312. doi: 10.1007/s00284-026-04880-8 (PMC13090207; doi:10.1007/s00284-026-04880-8)
Supplement: Supplementary file 1 — Supplementary file1 [file 284_2026_4880_MOESM1_ESM.docx]

**Supplementary material**

**^*^**Thandiwe Semumu^1, 2^, Nerve Zhou^2^, Kebaneilwe Lebani^2^, Thando Ndlovu^1^, Kabo Wale^1^ *Daniel Loeto^1^

*^1^Department of Biological Sciences, Faculty of Science, University of Botswana, Private Bag 0022 Gaborone, Botswana*

*^2^Department of Biological Sciences and Biotechnology, Faculty of Science, Botswana International University of Science and Technology, Private Bag 16 Palapye, Botswana*

*Corresponding author: Daniel Loeto: [loetod@ub.ac.bw](mailto:loetod@ub.ac.bw)

**S1**. Identity of yeast isolates from traditional sourdoughs around Botswana using the ITS region and their accession numbers. All sequences were last blasted on the 31st of July 2024

| **Sample name** | **Yeast isolate** | **Accession number** |  | **Repository accession number** |  |  |  |
| --- | --- | --- | --- | --- | --- | --- | --- |
| FJ1-A1 | *Saccharomyces cerevisiae* | CP006426.1 |  | PP864082 |  |  |  |
| FJ2-A1 | *Saccharomyces cerevisiae* | KY596697.1 |  | PP864080 |  |  |  |
| FJ2-A2 | *Wickerhamomyces anomalus*^aff^ | KT175191.1 |  | None |  |  |  |
| FJ2-A3 | *Saccharomyces cerevisiae* | CP006426.1 |  | PP864081 |  |  |  |
| GT1-A1 | *Saccharomyces cerevisiae*^aff^ | OP764057.1 |  | PP864083 |  |  |  |
| GA1-A1 | *Pichia kudriavzerii* | OP764057.1 |  | PP864074 |  |  |  |
| MN1-A1 | *Saccharomyces cerevisiae* | KY596697.1 |  | PP864084 |  |  |  |
| MN1-A2 | *Pichia membranifaciens* | KY495728.1 |  | PP864091 |  |  |  |
| MN1-A3 | *Saccharomyces cerevisiae* | KY596697.1 |  | PP864078 |  |  |  |
| GD1-A1 | *Saccharomyces cerevisiae* | KT958553.1 |  | PP864079 |  |  |  |
| GD1-A2 | *Pichia kudriavzevii* | CP039617.1 |  | PP864085 |  |  |  |
| GD1-A3 | *Pichia kudriavzevii* | KY457575.1 |  | PP864086 |  |  |  |
| GD1-A4 | *Saccharomyces cerevisiae* | KU500417.1 |  |  |  |  |  |
| TB1-A1 | *Saccharomyces cerevisiae* | KY495744.1 |  | PP864076 |  |  |  |
| TB1-A2 | *Saccharomyces cerevisiae* | LC576585.1 |  | PP864077 |  |  |  |
| RK1-A1 | *Saccharomyces cerevisiae* | CP006426.1 |  | PP864075 |  |  |  |
| RK1-A2 | *Saccharomyces cerevisiae*^aff^ | MW856066.1 |  | None |  |  |  |
| ME1-A1 | *Kazachstania unispora* | MK268124.1 |  | PP864089 |  |  |  |
| ME1-A2 | *Kazachstania unispora* | MK268124.1 |  | PP864090 |  |  |  |
| UK1-A1 | *Kazachstania humilis* | LS974436.1 |  | PP864087 |  |  |  |
| UK1-A2 | *Kazachstania humilis* | JQ726600.1 |  | PP864088 |  |  |  |
| Baker’s yeast | *Saccharomyces cerevisiae*^aff^ | KF447149.1 |  |  |  |  |  |

**S2.** Identity of lactic acid bacteria from tradition sourdoughs around Botswana using 16S region and their accession numbers. All sequences were last blasted on the 31^th^ of July 2024.

| **Sample name** | **Bacterium** | **Accession** |  | **Repository Accession number** |  |  |  |  |
| --- | --- | --- | --- | --- | --- | --- | --- | --- |
| GD1-B1 | *Lactiplantibacillus plantarum* | [MT463431.1](https://www.ncbi.nlm.nih.gov/nucleotide/MT463431.1?report=genbank&log$=nucltop&blast_rank=1&RID=3K4H9X40016) |  | PP860773 |  |  |  |  |
| GD1-B2 | *Lactiplantibacillus Plantarum* | [MT611900.1](https://www.ncbi.nlm.nih.gov/nucleotide/MT611900.1?report=genbank&log$=nucltop&blast_rank=1&RID=3JTAYTG5013) |  | PP860774 |  |  |  |  |
| GD1-B3 | *Lactiplantibacillus plantarum* | [CP052869.1](https://www.ncbi.nlm.nih.gov/nucleotide/CP052869.1?report=genbank&log$=nucltop&blast_rank=1&RID=3K3313J801N) |  | PP860775 |  |  |  |  |
| GD1-B4 | *Lactiplantibacillus plantarum* | [OR520803.1](https://www.ncbi.nlm.nih.gov/nucleotide/OR520803.1?report=genbank&log$=nucltop&blast_rank=2&RID=PBDP8DDD01N) |  | PP860791 |  |  |  |  |
| GA1-B1 | *Lacticaseibacillus paracasei* | [MT538398.1](https://www.ncbi.nlm.nih.gov/nucleotide/MT538398.1?report=genbank&log$=nucltop&blast_rank=1&RID=3K4YEVMZ013) |  | PP860776 |  |  |  |  |
| RK1-B4 | *Liquorilactobacillus nageli* | [ON705146.1](https://www.ncbi.nlm.nih.gov/nucleotide/ON705146.1?report=genbank&log$=nucltop&blast_rank=2&RID=PBDWR3Y101N) |  | PP860792 |  |  |  |  |
| FJ2-B3 | *Lacticaseibacillus paracasei* | AB759528.1 |  | PP860789 |  |  |  |  |
| ME1-B1 | *Lentilactobacillus parabuchneri* | ON125464.1 |  | PP860788 |  |  |  |  |
| ME1-B2 | *Liquorilactobacillus nageli* | [MT597696.1](https://www.ncbi.nlm.nih.gov/nucleotide/MT597696.1?report=genbank&log$=nucltop&blast_rank=1&RID=PBF3DAH001N) |  | PP860787 |  |  |  |  |
| GT1-B1 | *Lactiplantibacillus Plantarum* | [ON384540.1](https://www.ncbi.nlm.nih.gov/nucleotide/ON384540.1?report=genbank&log$=nucltop&blast_rank=4&RID=Y9KJAN3F013) |  |  |  |  |  |  |
|  |  |  |  |  |  |  |  |  |
|  |  |  |  |  |  |  |  |  |

**S3.** Identity of *Bacillus* and other bacteria from tradition sourdoughs around Botswana using 16S region and their accession numbers. All sequences were last blasted on the 31^th^ of July 2024.

| **Sample name** | **Bacillus and other bacteria** | **Accession** | **Repository Accession no.** |  |  |  |  |  |
| --- | --- | --- | --- | --- | --- | --- | --- | --- |
| GA1-B2 | *Bacillus cereus* | [ON740901.1](https://www.ncbi.nlm.nih.gov/nucleotide/ON740901.1?report=genbank&log$=nucltop&blast_rank=1&RID=3K634XYZ01N) |  | PP860777 |  |  |  |  |
| RK1-B1 | *Bacillus carboniphilus* | [KC494304.1](https://www.ncbi.nlm.nih.gov/nucleotide/KC494304.1?report=genbank&log$=nucltop&blast_rank=1&RID=3K7K5G1Z013) |  | PP860780 |  |  |  |  |
| RK1-B2 | *Bacillus siamensis* | [AB813716.1](https://www.ncbi.nlm.nih.gov/nucleotide/AB813716.1?report=genbank&log$=nucltop&blast_rank=1&RID=3K6V65NR016) |  | PP860778 |  |  |  |  |
| RK1-B3 | *Bacillus cereus* | [MK479915.1](https://www.ncbi.nlm.nih.gov/nucleotide/MK479915.1?report=genbank&log$=nucltop&blast_rank=1&RID=3R9V9139016) |  | PP860779 |  |  |  |  |
| FJ1-B1 | *Bacillus pumilus* | [KT970980.1](https://www.ncbi.nlm.nih.gov/nucleotide/KT970980.1?report=genbank&log$=nucltop&blast_rank=2&RID=3RA35K88016) |  | PP860781 |  |  |  |  |
| FJ2-B1 | *Bacillus zhangzhouensis* | [CP119300.1](https://www.ncbi.nlm.nih.gov/nucleotide/CP119300.1?report=genbank&log$=nucltop&blast_rank=2&RID=3SFS6Z7Y013) |  | PP860782 |  |  |  |  |
| FJ2-B2 | *Bacillus cereus* | [OP984781.1](https://www.ncbi.nlm.nih.gov/nucleotide/OP984781.1?report=genbank&log$=nucltop&blast_rank=1&RID=3RAKAW6T013) |  | PP860783 |  |  |  |  |
| MN1-B1 | *Bacillus stratosphericus* | [MG561355.1](https://www.ncbi.nlm.nih.gov/nucleotide/MG561355.1?report=genbank&log$=nucltop&blast_rank=3&RID=3RBRBDRU01N) |  | PP860784 |  |  |  |  |
| MN1-B2 | *Bhagavaea ginsengi* | [MN121192.1](https://www.ncbi.nlm.nih.gov/nucleotide/MN121192.1?report=genbank&log$=nucltop&blast_rank=1&RID=3RC81PMT013) |  | PP860785 |  |  |  |  |
| TB1-B1 | *Lysinibacillus halotolerans* | [OM142568.1](https://www.ncbi.nlm.nih.gov/nucleotide/OM142568.1?report=genbank&log$=nucltop&blast_rank=1&RID=3RC9171K016) |  | PP860786 |  |  |  |  |
| UK1-B1 | *Bacillus thuringiesis* | [OR741996.1](https://www.ncbi.nlm.nih.gov/nucleotide/OR741996.1?report=genbank&log$=nucltop&blast_rank=1&RID=Y9K1S81W016) |  | PP860793 |  |  |  |  |
| UK1-B2 | *Bacillus licheniformis* | MW282868.1 |  | PP860794 |  |  |  |  |

**S4.** Identity of Acetic acid bacteria from traditional sourdough around Botswana and their accession numbers. All sequences were last blasted on the 10th of June 2024

| **Sample name** | **Acetic acid bacteria** | **Accession** | **Repository Accession no.** |  |  |  |  |  |
| --- | --- | --- | --- | --- | --- | --- | --- | --- |
| GA1-C1 | *Acetobacter pasteurianus* | [MH845625.1](https://www.ncbi.nlm.nih.gov/nucleotide/MH845625.1?report=genbank&log$=nucltop&blast_rank=2&RID=PBRZZ50H016) | PP864057 |  |  |  |  |  |
| FJ1-C1 | *Acetobacter pasteurianus* | [EU096228.1](https://www.ncbi.nlm.nih.gov/nucleotide/EU096228.1?report=genbank&log$=nucltop&blast_rank=1&RID=PBSAPKHU016) | PP864057 |  |  |  |  |  |
| GD1-C1 | *Acetobacter indonesiensis* | [AB906412.1](https://www.ncbi.nlm.nih.gov/nucleotide/AB906412.1?report=genbank&log$=nucltop&blast_rank=1&RID=PBS7Y960016) | None |  |  |  |  |  |
| UK1-C1 | *Acetobacter malorum* | [EU096228.1](https://www.ncbi.nlm.nih.gov/nucleotide/EU096228.1?report=genbank&log$=nucltop&blast_rank=1&RID=PBRWX22U016) | PP864058 |  |  |  |  |  |

**Figure S5. Carbon assimilation of Yeast**

**Strain ID Maltose Raffinose Maltotriose Sucrose Glucose Fructose**

S. cerevisiae (BY)-control 0.6 ± 0.01 1.1 ± 0.01 0.1 ± 0.01 1.4 ± 0.01 1.4 ± 0.02 1.2 ± 0.02

S. cerevisiae (FJ2-A1) 1.2 ±0.01**** 0.8 ± 0.01 0.2 ± 0.01**** 1.4 ± 0.01 ns 1.3± 0.01 1.3 ± 0.01****

W. anomalus (FJ2-A2) 0.7 ± 0.01** 0.4 ± 0.01 0.4 ± 0.02**** 1.2 ± 0.02 1.2 ± 0.01 1.2 ± 0.00 ns

S. cerevisiae (FJ2-A3) 1.1 ± 0.02**** 0.7 ± 0.02 0.0 ± 0.00 1.5 ± 0.02 ns 1.3 ± 0.00 1.3 ± 0.01****

S. cerevisiae (FJ1-A1) 1.2 ± 0.00**** 0.5 ± 0.02 0.0 ± 0.01 1.5 ± 0.01 ns 1.3 ± 0.01 1.4 ± 0.0 ****

S. cerevisiae (MN1-A1) 0.1 ± 0.01 0.1 ± 0.00 0.1 ± 0.02 0.9 ± 0.02 0.8 ± 0.01 0.6 ± 0.02

P.membranifaciens 0.7 ± 0.01 0.5 ± 0.02 0.1 ± 0.01 1.4 ± 0.01 ns 1.2 ± 0.02 1.2 ± 0.01

S. cerevisiae (MN1-A3) 1.1 ± 0.01**** 0.6 ± 0.02 0.1 ± 0.02 1.4 ± 0.02 ns 1.4 ± 0.02 ns 1.4 ± 0.01****

S. cerevisiae (GD1-A1) 1.4 ± 0.03**** 1.5 ± 0.03**** 0.1 ± 0.02 1.5 ± 0.01**** 1.5 ± 0.01** 1.5 ± 0.01****

P. kudriavzerii (GD1-A2) 0.1 ± 0.01 0.2 ± 0.02 0.2 ± 0.03**** 0.4 ± 0.01 1.4 ± 0.02 ns 1.3 ± 0.01

P. kudriavzerii (GD1-A3) 0.1 ± 0.02 0.2 ± 0.00 0.1 ± 0.01 0.4 ± 0.01 0.9 ± 0.01 0.9 ± 0.01

P. kudriavzerii (GD1-A4) 0.2 ± 0.01**** 0.3 ± 0.01 0.2 ± 0.02**** 0.1 ± 0.00 0.8 ± 0.01 1.3 ± 0.01

P. kudriavzerii (GA1-A1) 1.1 ± 0.02**** 1.4 ± 0.01**** 0.3 ± 0.01**** 1.4 ± 0.02 ns 1.4 ± 0.02 ns 1.4 ± 0.01****

S. cerevisiae (RK1-A1) 1.2 ± 0.01**** 0.1 ± 0.01 0.1 ± 0.02 0.9 ± 0.01 1.3 ± 0.02 1.1 ± 0.02

S. cerevisiae (RK1-A2) 0.7 ± 0.01 0.1 ± 0.02 0.4 ± 0.01**** 0.1 ± 0.01 1.2 ± 0.01 1.1 ± 0.01

K. unispora (ME1-A1) 0.9 ± 0.01**** 0.6 ± 0.02 0.1 ± 0.00 1.4 ± 0.01 ns 1.3 ± 0.02 1.3 ± 0.01

K. unispora (ME1-A2) 0.1 ± 0.01 1.1 ± 0.02 0.1 ± 0.01 1.3 ± 0.02 1.2 ± 0.02 1.3 ± 0.01

S. cerevisiae (TB1-A1) 0.1 ± 0.02 0.2 ± 0.02 0.2 ± 0.03**** 0.1 ± 0.01 1.3 ± 0.01 1.4 ± 0.01****

S. cerevisiae (TB1-A2) 0.1 ± 0.01 0.2 ± 0.01 0.1 ± 0.01 0.1 ± 0.01 1.4 ± 0.01 ns 1.4 ± 0.02****

K. humilis (UK1-A1) 1.4 ± 0.02**** 1.6 ± 0.02**** 0.1 ± 0.02 1.5 ± 0.01**** 1.5 ± 0.01* 1.5 ± 0.01****

C. humilis (UK1-A2) 1.1 ± 0.03**** 1.5 ± 0.01**** 0.2 ± 0.02**** 1.4 ± 0.01 ns 1.4 ± 0.01ns 1.4 ± 0.02****

* The data represent the mean value (± the SEM) from three Biological replicates. Significant differences between samples and their corresponding control (BY) is denoted with *, **, ***, **** indicating significance, p < 0.05, < 0.01, < 0.001, and < 0.0001 respectively.

**Figure S6. Carbon assimilation of Bacteria**

**Strain ID Maltose Raffinose Maltotriose Sucrose Glucose Fructose**

S. cerevisiae (BY)-control 0.64 ± 0.01 1.07 ±0.02 0.14 ± 0.01 1.42 ± 0.01 1.40 ± 0.02 1.18 ± 0.02

B. zhangzhouensis (FJ2-B1) 0.35 ± 0.01 0.44 ±0.01 0.40 ± 0.01**** 0.38 ± 0.02 0.58 ± 0.01 0.33 ± 0.01

B. cereus (FJ2-B2) 0.03 ± 0.01 0.07± 0.03 0.05 ± 0.01 0.07 ± 0.01 0.05 ± 0.00 0.15 ± 0.01

L. paracasei (FJ2-B3) 0.00 ± 0.02 0.00± 0.02 0.00 ± 0.03 0.05 ± 0.01 0.04 ± 0.02 0.05 ± 0.02

B. pumilis(FJ1-B1) 0.08 ±0.02 0.05± 0.03 0.04 ± 0.00 0.16 ± 0.01 0.02 ± 0.02 0.12 ± 0.01

B. stratosphericus (MN1-B1) 0.05 ± 0.02 0.04± 0.03 0.02 ± 0.01 0.03 ± 0.01 0.04 ± 0.00 0.14 ± 0.01

B. ginseng (MN1-B2) 0.01 ± 0.00 0.39± 0.02 0.07 ± 0.01 0.03 ± 0.02 0.07 ± 0.02 0.14 ± 0.01

L. plantarum (GD1-B1) 0.04 ± 0.01 0.04± 0.03 0.06 ± 0.01 0.07 ± 0.02 0.05 ± 0.01 0.14 ± 0.01

L. plantarum (GD1-B2) 0.10 ± 0.01 0.08± 0.03 0.08 ± 0.01 0.09 ± 0.01 0.11 ± 0.01 0.08 ± 0.01

L. plantarum (GD1-B3) 0.01 ± 0.01 0.01± 0.01 0.00 ± 0.02 0.04 ± 0.02 0.04 ± 0.01 0.14 ± 0.01

L. paracasei (GA1-B1) 0.02 ± 0.02 0.01± 0.03 0.02 ± 0.01 0.01 ± 0.02 0.06 ± 0.01 0.07 ± 0.02

B. cereus (GA1-B2) 0.01 ± 0.01 0.00± 0.03 0.03 ± 0.01 0.00 ± 0.01 0.00 ± 0.02 0.12 ± 0.02

B. carboniphilus (RK1-B1) 0.05 ± 0.01 0.04± 0.03 0.05 ± 0.02 0.06 ± 0.01 0.05 ± 0.01 0.12 ± 0.03

B. siamensis (RK1-B2) 0.05 ± 0.01 0.01± 0.02 0.00 ± 0.01 0.12 ± 0.02 0.05 ± 0.01 0.14 ± 0.02

B. cereus (RK1-B3) 0.01 ± 0.00 0.33± 0.04 0.02 ± 0.01 0.03 ± 0.01 0.04 ± 0.00 0.08 ± 0.01

L. nageli (RK1-B4) 0.01 ± 0.01 0.00± 0.04 0.00 ± 0.00 0.01 ± 0.01 0.00 ± 0.01 0.12 ± 0.02

L. platarum (ME1-B1) 0.01 ± 0.02 0.00± 0.03 0.01 ± 0.01 0.18 ± 0.01 0.00 ± 0.01 0.07 ± 0.00

L. nageli (ME1-B2) 0.40 ± 0.02 0.33± 0.04 0.21 ± 0.02* 0.33 ± 0.02 0.12 ± 0.02 0.31 ± 0.02

L. halotolerans (TB1-B1) 0.07 ± 0.01 0.06± 0.01 0.06 ± 0.01 0.05 ± 0.03 0.08 ± 0.02 0.16 ± 0.01

B.cereus (GT1-B1) 0.02 ± 0.01 0.01± 0.02 0.00 ± 0.01 0.07 ± 0.02 0.09 ± 0.03 0.02 ± 0.01

B. thuringiensis (UK1-B1) 0.00 ± 0.01 0.00± 0.02 0.00 ± 0.02 0.01 ± 0.01 0.00 ± 0.01 0.19 ± 0.01

B.licheniformis (UK1-B2) 0.27 ± 0.01 0.25± 0.02 0.24 ± 0.01* 0.27 ± 0.01 0.26 ± 0.02 0.38 ± 0.01

A. pasteurianus (GA1-C1) 0.01 ± 0.01 0.01± 0.04 0.00 ± 0.01 0.05 ± 0.02 0.00 ± 0.01 0.13 ± 0.02

A.pasteurianus (FJ1-C1) 0.00 ± 0.01 0.00± 0.02 0.06 ± 0.01 0.01 ± 0.01 0.00 ± 0.02 0.12 ± 0.00

A.indonesiensis (GD1-C1) 0.77 ± 0.01*** 0.47± 0.02 0.52 ± 0.01**** 0.63 ± 0.01 0.67 ± 0.01 0.39 ± 0.01

A.malorum (UK1-C1) 0.20 ± 0.01 0.12± 0.01 0.26 ± 0.01* 0.17 ± 0.00 0.17 ± 0.01 0.03 ± 0.01

* The data represent the mean value (± the SEM) from three Biological replicates. Significant differences between samples and their corresponding control (BY) is denoted with *, **, ***, **** indicating significance, p < 0.05, < 0.01, < 0.001, and < 0.0001 respectively.

**S7**; Carbon dioxide production rate of isolated yeasts using different carbon sources.

| Strain Strain ID | Maltose | Glucose | Fructose | Sucrose | Synthetic dough |
| --- | --- | --- | --- | --- | --- |
| *S. cerevisiae* (FJ2-A1) | 0.3608±0.02 | 0.3138±0.06 | 0.4304±0.05 | 0.2327±0.4 | 0.4024±6.7E-17 |
| *W. anomalus* (FJ2-A2) | 0.0726±0.03 | 0.4607±0.02 | 0.3598±0.07 | 0.2753±0.1 | 0±0 |
| *S. cerevisiae* (FJ2-A3) | 0.3131±0.04 | 0.4259±0.09 | 0±0 | 0.2118±0 | 0.1837±0.05 |
| *S. cerevisiae* (FJ1-A1) | 0.4304±0.05 | 0.3619±0.02 | 0±0 | 0±0 | 0±0 |
| *S. cerevisiae* (MN1-A1) | 0.3027±0.05 | 0.3348±0.04 | 0.4368±0.9 | 0±0 | 0.2118±0 |
| *P. membranifaciens* (MN1-A2) | 0.2747±0 | 0±0 | 0±0 | 0±0 | 0±0 |
| *S. cerevisiae* (MN1-A3) | 0.3103±0.04 | 0.3464±0.08 | 0.4304±0.05 | 0.0425±0.07 | 0.4865±0 |
| *S. cerevisiae* (GD1-A1) | 0.28716±0.06 | 0.3774±0.05 | 0±0 | 0.2118±0 | 0.2665±0.05 |
| *P. kudriavzerii* (GD1-A2) | 0.1581±0.03 | 0.4287 | 0.4024±6.7E-17 | 0±0 | 0±0 |
| *P. kudriavzerii* (GD1-A3) | 0±0 | 0.5074±0.04 | 0.2963±0.09 | 0±0 | 0±0 |
| *P. kudriavzerii* (GD1-A4) | 0±0 | 0.2507±0.04 | 0.2747±0 | 0±0 | 0±0 |
| *P. kudriavzerii* (GA1-A1) | 0.3051±0.05 | 0.3935±0.05 | 0±0 | 0.2118±0 | 0.2456±0.06 |
| *S. cerevisiae* (RK1-A1) | 0.2675±0.05 | 0.2827±0.03 | 0±0 | 0±0 | 0.4024±6.7E-17 |
| *S. cerevisiae* (RK1-A2) | 0.5283±0.04 | 0.2879±0.04 | 0.4304±0.05 | 0.2118±0 | 0.4024±6.7E-17 |
| *K. unispora* (ME1-A1) | 0.2747±0 | 0.1891±0.01 | 0.3081±0.03 | 0±0 | 0.3598±0.07 |
| *K. unispora* (ME1-A2) | 0.2834±0.02 | 0.2229±0.01 | 0.2507±0.04 | 0±0 | 0.4024±6.7E-17 |
| *S. cerevisiae* (TB1-A1*)* | 0.1277±0 | 0.2071±0.08 | 0.2199±0.02 | 0±0 | 0.2118±0 |
| *S. cerevisiae* (TB1-A2) | 0.1277±0 | 0.2017±0.03 | 0.2118±0 | 0±0 | 0.4024±6.7E-17 |
| *K. humilis* (UK1-A1) | 0±0 | 0.3464±0.01 | 0.2537±0.04 | 0.2118±0 | 0.2747±0 |
| *C. humilis* (UK1-A2) | 0±0 | 0­±0 | 0.2997±0.04 | 0.2118±0 | 0.2747±0 |
| *S. cerevisiae* (BY) | 0.3884±0.01 | 0.2641±0.05 | 0.1557±0.05 | 0.2118±0 | 0.3388±0.1 |

**S8:** Fermentation capability of bacteria from Traditional sourdough of Botswana

| Strain ID | Maltose | Glucose | Fructose | Sucrose | Synthetic dough |
| --- | --- | --- | --- | --- | --- |
| *B. zhangzhouensis* (FJ2-B1) | **-** | **-** | **-** | **-** | **-** |
| *B. cereus* (FJ2-B2) | **+** | **-** | **-** | **-** | **-** |
| *L. paracasei* (FJ2-B3) | **+** | **+** | **+** | **-** | **-** |
| *B. pumilis* (FJ1-B1) | **+** | **+** | **-** | **+** | **-** |
| *L. paracasei* (GA1-B1) | **+** | **+** | **+** | **-** | **-** |
| *B. cereus* (GA1-B2) | **+** | **-** | **-** | **-** | **-** |
| *L. plantarum* (ME1-B1) | **+** | **+** | **-** | **-** | **+** |
| *L. nageli* (ME1-B2) | **+** | **-** | **+** | **-** | **+** |
| *L. halototerans* (TB1-B1) | **-** | **-** | **-** | **-** | **-** |
| *L. plantarum* (GD1-B1) | **+** | **+** | **-** | **-** | **+** |
| *L. plantarum* (GD1-B2) | **+** | **+** | **-** | **-** | **-** |
| *L. plantarum* (GD1-B3) | **+** | **+** |  |  | **+** |
| *B. stratosphericus* (MN1-B1) | **-** | **-** | **-** | **-** | **-** |
| *B. gingseng* (MN1-B2) | **-** | **-** | **-** | **-** | **-** |
| *B. carboniphilus* (RK1-B1) | **-** | **+** | **-** | **-** | **-** |
| *B. siamensis* (RK1-B2) | **-** | **+** | **-** | **-** | **-** |
| *B. cereus* (RK1-B3) | **+** | **-** | **-** | **-** | **-** |
| *L. nageli* (RK1-B4) | **+** | **-** | **+** | **-** | **+** |
| *B. cereus* (GT1-B1) | **+** | **-** | **-** | **-** | **-** |
| *B. thuringiensis* (UK1-B1) | **+** | **+** | **+** | **-** | **-** |
| *B. licheniformis* (UK1-B2) | **-** | **+** | **-** | **-** | **-** |

(++: enough CO_2_ to lift the plunger, +: bubbles produced but could not lift plunger, -: could not produce carbon dioxide)

**S9**: Growth profiles of isolated yeasts and bacteria subjected to different baking associated stressors.

| Strain ID | 1 mM H_2_O_2_ | 2 mM H_2_O_2_ | 3 mM H_2_O_2_ | 4 mM H_2_O_2_ | 5 mM H_2_O_2_ | 2% C_2_H_5_OH | 4% C_2_H_5_OH | 6% C_2_H_5_OH | 8% C_2_H_5_OH | 10.% C_2_H_5_OH | 1%CH_3_OOH | 1%C_3_H_6_O_3_ | 0.5 M NaCl | 0.75 M NaCl | 1.0 M NaCl | 1.25 M NaCl | 1.5 M NaCl | 25°C | 30.°C | 35°C | 37°C | 42°C | pH 3 | pH 3.5 | pH 4.0 |
| --- | --- | --- | --- | --- | --- | --- | --- | --- | --- | --- | --- | --- | --- | --- | --- | --- | --- | --- | --- | --- | --- | --- | --- | --- | --- |
| *S. cerevisiae (FJ2-A1)* | 0.178 | 0.092333 | 0.32 | 0.094 | 0.097333 | 0.093667 | 0.09 | 0.094 | 0.148667 | 0.043333 | 0.338833 | 0.02 | 0.109 | 0.112667 | 0.141667 | 0.112667 | 0.144667 | 0.827667 | 0.829667 | 0.614 | -0.15633 | 0.133667 | 0.852667 | 0.808667 | 0.737333 |
| *W. anomalus (FJ2-A2)* | 0.223333 | 0.063333 | 0.043 | 0.131667 | 0.085 | 0.075333 | 0.12 | 0.261 | 0.177333 | 0.100333 | 0.045333 | 0.019333 | 0.177667 | 0.173333 | 0.127333 | 0.135333 | 0.222333 | 0.902667 | 0.930333 | 0.958 | 0.839 | 0.011 | 0.985333 | 1.107 | 1.099667 |
| *S. cerevisiae (FJ2-A3)* | 0.131 | 0.144667 | 0.076333 | 0.108 | 0.133667 | 0.108333 | 0.078667 | 0.141 | 0.102667 | 0.09 | 0.221167 | 0.02 | 0.081333 | 0.123333 | 0.105667 | 0.079667 | 0.127333 | 0.847333 | 0.703 | 0.761667 | 0.768 | 0.276667 | 0.673333 | 0.775333 | 0.824 |
| *S. cerevisiae (FJ1-A1)* | 0.135667 | 0.066333 | 0.063667 | 0.136333 | 0.107333 | 0.101667 | 0.067333 | 0.149 | 0.094 | 0.16 | 0.106167 | 0.019333 | 0.077667 | 0.057 | 0.082 | 0.034333 | 0.116667 | 0.864 | 0.668333 | 0.799333 | 0.789667 | 0.248 | 0.763 | 0.79 | 0.862667 |
| *S. cerevisiae (MN1-A1)* | 0.147333 | 0.128667 | 0.082333 | 0.112333 | 0.231333 | 0.072667 | 0.169 | 0.039667 | 0.158667 | 0.043667 | 0.371167 | 0.027 | 0.143667 | 0.101667 | 0.081 | 0.064 | 0.055 | 0.848 | 0.773333 | 0.751667 | 0.746333 | 0.194667 | 0.871667 | 1.041333 | 0.919 |
| *P.membranifaciens(MN1-A2)* | 0.098667 | 0.086 | 0.056333 | 0.059333 | 0.053 | 0.067 | 0.089667 | 0.073 | 0.113 | 0.043667 | 0.212833 | 0.023333 | 0.087667 | 0.063667 | 0.076333 | 0.052 | 0.063667 | 0.820333 | 0.498333 | 0.799 | 0.872 | 0.114 | 0.722667 | 0.896667 | 0.884 |
| *S. cerevisiae (MN1-A3)* | 0.118 | 0.136 | 0.156667 | 0.159 | 0.113333 | 0.121 | 0.139333 | 0.167333 | 0.189333 | 0.076333 | 0.529833 | 0.051333 | 0.135333 | 0.138333 | 0.091667 | 0.157 | 0.155 | 0.801333 | 0.699667 | 0.717333 | 0.781333 | 0.205667 | 0.809 | 0.943667 | 0.849333 |
| *S. cerevisiae (GD1-A1)* | 0.202333 | 0.206333 | 0.251333 | 0.161333 | 0.162 | 0.187667 | 0.243 | 0.352 | 0.179667 | 0.145 | 0.435167 | 0.030333 | 0.118333 | 0.148667 | 0.085 | 0.141667 | 0.157333 | 0.499667 | 0.433333 | 0.749667 | 0.331 | 0.358333 | 0.907667 | 1.022667 | 1.014667 |
| *P. kudriavzerii (GD1-A2)* | 0.127 | 0.147 | 0.111667 | 0.101 | 0.096333 | 0.145667 | 0.09 | 0.070667 | 0.104667 | 0.107667 | 1.115 | 0.014 | 0.107667 | 0.121667 | 0.144667 | 0.134667 | 0.17 | 0.849667 | 0.763667 | 0.715 | 0.241667 | 0.235333 | 0.776667 | 1.064667 | 0.996667 |
| *P. kudriavzerii (GD1-A3)* | 0.085 | 0.115333 | 0.081 | 0.088333 | 0.086667 | 0.087 | 0.086 | 0.082667 | 0.073667 | 0.091333 | 1.085333 | 0.013333 | 0.094667 | 0.089333 | 0.104 | 0.074667 | 0.122667 | 0.867667 | 0.782333 | 0.841333 | 0.813667 | 0.696333 | 0.886667 | 0.812667 | 0.827667 |
| *P. kudriavzerii (GD1-A4)* | 0.071 | 0.077667 | 0.060333 | 0.058333 | 0.06 | 0.068333 | 0.071 | 0.073667 | 0.065 | 0.072 | 1.111333 | 0.018333 | 0.065667 | 0.075 | 0.104333 | 0.076 | 0.105667 | 0.927667 | 0.919667 | 0.926333 | 0.873 | 0.495 | 1.018 | 1.076333 | 1.013 |
| *P. kudriavzerii (GA1-A1)* | 0.164667 | 0.163333 | 0.096667 | 0.273333 | 0.171667 | 0.175667 | 0.318 | 0.311333 | 0.157 | 0.170333 | 0.453833 | 0.029333 | 0.111333 | 0.109333 | 0.145 | 0.144667 | 0.099333 | 0.814333 | 0.784 | 0.850333 | 0.782667 | 0.211667 | 0.818333 | 0.95 | 0.898 |
| *S. cerevisiae (RK1-A1)* | 0.113333 | 0.121667 | 0.098333 | 0.090667 | 0.091 | 0.091333 | 0.159333 | 0.114667 | 0.118667 | 0.06 | 0.2115 | 0.041333 | 0.075 | 0.082 | 0.1 | 0.079667 | 0.094333 | 0.793667 | 0.667 | 0.721333 | 0.692667 | -0.03933 | 0.787667 | 0.851333 | 0.877667 |
| *S. cerevisiae (RK1-A2)* | 0.131667 | 0.106333 | 0.112333 | 0.109 | 0.125 | 0.114 | 0.159 | 0.090667 | 0.098333 | 0.074667 | 0.364833 | 0.039667 | 0.099 | 0.101 | 0.072667 | 0.117333 | 0.098333 | 0.708 | 0.638 | 0.623667 | 0.703333 | -0.055 | 0.759667 | 0.836 | 0.749333 |
| *K. unispora (ME1-A1)* | 0.105667 | 0.113 | 0.115667 | 0.124333 | 0.096 | 0.097 | 0.117333 | 0.135 | 0.141 | 0.11 | 0.1805 | 0.034 | 0.111 | 0.098 | 0.111667 | 0.102667 | 0.13 | 0.807 | 0.702667 | 0.759333 | 0.774333 | 0.290667 | 0.881333 | 0.964 | 0.883667 |
| *K. unispora (ME1-A2)* | 0.104333 | 0.114333 | 0.114333 | 0.154333 | 0.124667 | 0.087 | 0.143667 | 0.176 | 0.139 | 0.204333 | 0.011333 | 0.012 | 0.105667 | 0.084 | 0.081667 | 0.097 | 0.093667 | 0.634 | 0.654333 | 0.296 | 0.004333 | -0.03767 | 0.566 | 0.95 | 1.004 |
| *S. cerevisiae (TB1-A1)* | 0.186333 | 0.17 | 0.122333 | 0.159333 | 0.205 | 0.087333 | 0.111333 | 0.067667 | 0.148667 | 0.045667 | 0.166 | 0.065667 | 0.126667 | 0.129667 | 0.153667 | 0.138667 | 0.171667 | 0.765667 | 0.756 | 0.280333 | -0.29467 | -0.17633 | 0.600333 | 0.883 | 0.843667 |
| *S. cerevisiae (TB1-A2)* | 0.180333 | 0.136333 | 0.177333 | 0.133333 | 0.138667 | 0.126667 | 0.124333 | 0.178667 | 0.115 | 0.074667 | 0.147667 | 0.056333 | 0.120667 | 0.102333 | 0.109667 | 0.130667 | 0.140333 | 0.790333 | 0.750667 | 0.675667 | 0.632667 | 0.050667 | 0.835667 | 0.909333 | 0.856667 |
| *K. humilis (UK1-A1)* | 0.197667 | 0.306333 | 0.207333 | 0.245667 | 0.212667 | 0.142333 | 0.217667 | 0.207667 | 0.151667 | 0.109667 | 0.295667 | 0.019667 | 0.144667 | 0.132 | 0.275333 | 0.154 | 0.183333 | 0.826 | 0.618 | 0.659333 | 0.689667 | -0.04533 | 1.029 | 1.079 | 0.956333 |
| *C. humilis (UK1-A2)* | 0.157333 | 0.239333 | 0.152333 | 0.271 | 0.214 | 0.099333 | 0.19 | 0.234667 | 0.158333 | 0.117333 | 0.365167 | 0.033 | 0.103 | 0.118333 | 0.115333 | 0.127 | 0.115 | 0.737667 | 0.756333 | 0.795667 | 0.756 | 0.066667 | 0.968667 | 1.047667 | 0.941333 |
| *S. cerevisiae (BY)* | 0.071333 | 0.099667 | 0.042333 | 0.071333 | 0.079 | 0.050667 | 0.073333 | 0.08 | 0.085 | 0.096 | 0.136167 | 0.018333 | 0.055333 | 0.057667 | 0.068 | 0.058 | 0.072 | 0.908333 | 1.108 | 1.09 | 0.83 | 0.031333 | 0.598667 | 0.750667 | 0.635667 |
| *B. zhangzhouensis (FJ2-B1)* | -0.005 | 0.002333 | 0.004333 | -0.00367 | -0.021 | 0.002 | -0.00833 | -0.008 | -0.001 | 0.001333 | 0.042333 | -0.001 | 0.006667 | 0.004667 | -0.00267 | 0.006333 | 0.010333 | -0.00433 | -0.00233 | 0.000667 | 0.002667 | 0.003333 |  |  |  |
| *B. cereus (FJ2-B2)* | -0.00233 | 0.001333 | 0 | 0.007333 | 0.021333 | 0.004667 | -0.00233 | -0.003 | 0.133333 | 0.011667 | 0.054333 | 0.044667 | -0.008 | 0.005667 | -0.00933 | 0.004333 | 0.063667 | 0.038667 | 0.010667 | 0.028 | 0.052667 | -0.01 |  |  |  |
| *L. paracasei (FJ2-B3)* | 0.004667 | -0.008 | -0.00967 | -0.001 | 0.009667 | 0.000333 | -0.002 | -0.004 | -0.00267 | -0.01067 | -0.00733 | -0.00767 | -0.004 | -0.006 | -0.00733 | -0.00233 | 0.002 | -0.00433 | -0.00767 | -0.00333 | 0.002 | -0.004 |  |  |  |
| *B. pumilis(FJ1-B1)* | 0.022667 | 0.000333 | -0.02133 | 0.015 | -0.00067 | 0.027333 | 0.011 | 0.010333 | -0.00533 | 0.007 | 0.006667 | 0.008667 | 0.003667 | 0.010667 | -0.01567 | 0.008667 | 0.023667 | 0.002667 | -0.00333 | 0.006 | 0.007667 | 0.000333 |  |  |  |
| *B. stratosphericus (MN1-B1)* | -0.001 | -0.00733 | -0.00367 | 0.028333 | -0.00733 | 0.017 | 0.000333 | -0.00433 | 0.000333 | 0.002 | -0.01 | -0.00767 | 0.004667 | 0.006 | 0.000333 | 0.049333 | 0.015667 | -0.00533 | 0.002 | 0.009 | 0.001333 | 0.006 |  |  |  |
| *B. ginseng (MN1-B2)* | 0.242 | 0.094333 | 0.328 | 0.334667 | 0.337667 | 0.196667 | 0.212 | 0.202667 | 0.118667 | 0.169667 | 0.124 | 0.119333 | -0.01633 | 0.152 | 0.037 | 0.058333 | 0.133333 | 0.202667 | 0.277667 | 0.201 | 0.188 | 0.250667 |  |  |  |
| *L. plantarum (GD1-B1)* | -0.01267 | -0.015 | 0.152333 | -0.00167 | 0.004333 | 0.007333 | 0.007 | -0.003 | 0.002333 | -0.00567 | 0.006 | -0.01267 | -0.01267 | -0.016 | -0.00267 | -0.013 | 0.002 | 0.034 | -0.001 | -0.00433 | 0.017 | -0.012 |  |  |  |
| *L. plantarum (GD1-B2)* | -0.013 | -0.00333 | 0.013333 | 0.011333 | 0.011 | 0.007 | 0.015 | -0.003 | 0.002667 | 0.002 | 0.04 | 0.043 | 0.007 | 0.009667 | 0.009333 | 0.056333 | 0.056667 | 0.002 | 0.001667 | 0.005333 | 0.002 | -0.00167 |  |  |  |
| *L. plantarum (GD1-B3)* | 0.000667 | -0.00067 | -0.013 | -0.006 | -0.01733 | 0.010667 | -0.015 | -0.01267 | -0.00167 | -0.00467 | 0.001 | 0.01 | 0.005667 | 0.004 | -0.00467 | 0.005667 | 0.008667 | 0.013667 | 0.002333 | 0.000667 | -0.00267 | -0.015 |  |  |  |
| *L. paracasei (GA1-B1)* | -0.01433 | -0.00767 | -0.023 | 0.026333 | 0.026 | 0.005333 | -0.001 | -0.00933 | 0.024667 | -0.007 | -0.021 | -0.01667 | -0.01033 | -0.00433 | -0.01167 | -0.00133 | -0.001 | -0.00967 | -0.005 | 0.038 | -0.012 | -0.01833 |  |  |  |
| *B. cereus (GA1-B2)* | 0.005 | -0.003 | -0.00667 | 0.002 | -0.019 | 0.007333 | -0.003 | -0.00133 | -0.01067 | -0.00233 | -0.00333 | -0.00267 | -0.00567 | -0.00267 | -0.013 | 0.003 | 0.008667 | -0.00333 | -0.00067 | 0.048667 | -0.005 | -0.00567 |  |  |  |
| *B. carboniphilus (RK1-B1)* | 0.085333 | 0.070333 | 0.045667 | 0.102667 | 0.035333 | 0.006 | -0.00133 | 0.005667 | 0.055 | -0.00033 | 0.026667 | 0.016 | -0.00533 | 0.013333 | 0.014333 | 0.016667 | 0.006333 | 0.055667 | 0.042333 | 0.041333 | 0.005667 | -0.014 |  |  |  |
| *B. siamensis (RK1-B2)* | -0.00467 | -0.00033 | -0.01 | 0.005333 | 0.059 | -0.00267 | -0.00433 | 0.026 | -0.00533 | -0.00367 | -0.01433 | 0.001667 | -0.025 | -0.05367 | -0.00067 | -0.049 | 0.009667 | -0.04933 | 0.1 | -0.003 | -0.05067 | 0.044 |  |  |  |
| *B. cereus (RK1-B3)* | 0.012667 | -0.01567 | 0.000667 | 0.005333 | -0.03867 | 0.013333 | 0.027667 | 0.009 | 0.005 | 0.011667 | 0.000667 | 0.004667 | 0.013667 | -0.004 | -0.021 | 0.001667 | 0.014333 | 0.003667 | 0.011 | 0.003333 | -0.008 | -0.00433 |  |  |  |
| *L. nageli (RK1-B4)* | 0.015 | 0.011 | 0.025 | 0.013333 | 0.03 | 0.012 | 0.001 | 0.009667 | 0.037667 | 0.054667 | 0.028667 | 0.022667 | 0.030333 | 0.036667 | 0.029 | 0.065 | 0.09 | 0.021667 | 0.020667 | 0.014 | 0.009667 | 0.001333 |  |  |  |
| *L. platarum (ME1-B1)* | 0.005667 | -0.00133 | -0.00167 | -0.004 | -0.03933 | -0.001 | 0.002667 | -0.002 | 0.042333 | 0.017 | 0.022 | 0.015667 | 0.007667 | 0.042 | 0.011667 | 0.042667 | 0.009333 | 0.002667 | 0.003333 | 0.004667 | 0.008 | -0.00567 |  |  |  |
| *L. nageli (ME1-B2)* | 0.228 | 0.197667 | 0.188667 | 0.295 | 0.355333 | 0.178333 | 0.242667 | 0.181 | 0.103667 | 0.035667 | 0.195333 | 0.075333 | 0.000667 | 0.067 | 0.053667 | -0.10233 | -0.08733 | 0.165 | 0.188333 | 0.253 | 0.339667 | 0.085333 |  |  |  |
| *L. halotolerans (TB1-B1)* | 0.001 | -0.006 | 0.033333 | -0.00167 | -0.03033 | 0.027 | -0.00467 | -0.009 | -0.01067 | -0.00067 | -0.002 | -0.00267 | 0.000667 | 0.005333 | -0.01767 | -0.00033 | 0.004333 | -0.001 | 0.001667 | 0.001667 | 0.001667 | -0.00167 |  |  |  |
| *B.cereus (GT1-B1)* | -0.00333 | -0.00133 | -0.004 | 0.001 | 0.012667 | 0.002333 | 0.000333 | -0.00333 | 0.008333 | 0.006 | -0.00067 | -0.01133 | 0.009 | 0.002333 | -0.002 | 0.05 | 0.047333 | 0.000667 | 0.000667 | 0.005 | 0 | -0.00867 |  |  |  |
| *B. thuringiensis (UK1-B1)* | 0.003667 | -0.001 | -0.00133 | 0.005333 | -0.18767 | 0.025667 | -0.00067 | -0.01067 | -0.00533 | 0.001333 | 0.000667 | 0.024667 | 0.002333 | -0.00067 | -0.01967 | 0.026 | 0.028 | 0.002333 | -0.00167 | 0.002333 | 0 | -0.00433 |  |  |  |
| *B.licheniformis (UK1-B2)* | 0.021667 | 0.007667 | 0.018 | 0.016667 | 0.006667 | 0.016333 | 0.035333 | 0.017 | 0.045 | 0.014333 | 0.042333 | 0.051333 | 0.013 | 0.050333 | 0.014667 | 0.052667 | 0.055333 | 0.010333 | 0.012667 | 0.020333 | 0.038667 | 0.012667 |  |  |  |
| *A. pasteurianus (GA1-C1)* | -0.003 | -0.00133 | -0.015 | 0.034333 | -0.02267 | 0.003333 | 0 | 0.003333 | -0.00667 | -0.00567 | -0.00367 | -0.00433 | -0.032 | -0.01033 | -0.011 | -0.009 | -0.007 | -0.00433 | -0.012 | -0.005 | 0.004333 | -0.00467 |  |  |  |
| *A.pasteurianus (FJ1-C1)* | 0.004667 | 0.000333 | -0.005 | -0.02167 | 0.042333 | 0.002667 | 0.004 | 0.005 | 0.007 | -0.00567 | 0.004 | -0.006 | -0.00033 | -0.00467 | -0.01333 | 0.021333 | 0.012333 | -0.00533 | -0.007 | 0.010667 | 0.008333 | -0.004 |  |  |  |
| *A.indonesiensis (GD1-C1)* | -0.014 | -0.01733 | -0.02033 | -0.00867 | 0.025333 | -0.00167 | -0.00867 | -0.006 | -0.01033 | -0.014 | -0.00033 | -0.003 | -0.02067 | -0.013 | -0.021 | -0.014 | 0.029 | -0.01267 | -0.01333 | -0.00933 | -0.00633 | -0.00967 |  |  |  |
| *A.malorum (UK1-C1)* | 0.006 | -0.01467 | -0.00367 | -0.00167 | 0.031333 | 0.003 | 0.003 | 0 | 0.003 | -0.01267 | 0.000333 | 0.024 | -0.007 | -0.006 | -0.004 | 0.002333 | 0.002333 | 0.002667 | -0.00467 | 0.000333 | 0.003667 | -0.004 |  |  |  |
